# Change in the Kinetic Regime of Aggregation of Yeast Alcohol Dehydrogenase in the Presence of 2-Hydroxypropyl-β-cyclodextrin

**DOI:** 10.3390/ijms242216140

**Published:** 2023-11-09

**Authors:** Vera A. Borzova, Andrey M. Chernikov, Valeriya V. Mikhaylova, Boris I. Kurganov

**Affiliations:** Bach Institute of Biochemistry, Federal Research Centre “Fundamentals of Biotechnology” of the Russian Academy of Sciences, Leninsky pr. 33, 119071 Moscow, Russia; chernikov.andrei.m@gmail.com (A.M.C.); mikhaylova.inbi@inbox.ru (V.V.M.);

**Keywords:** yeast alcohol dehydrogenase, protein denaturation, protein aggregation, aggregation kinetics, chemical chaperones, 2-hydroxypropyl-β-cyclodextrin

## Abstract

Chemical chaperones are low-molecular-weight compounds that suppress protein aggregation. They can influence different stages of the aggregation process—the stage of protein denaturation, the nucleation stage and the stage of aggregate growth—and this may lead to a change in the aggregation kinetic regime. Here, the possibility of changing the kinetic regime in the presence of a chemical chaperone 2-hydroxypropyl-β-cyclodextrin (2-HP-β-CD) was investigated for a test system based on the thermally induced aggregation of yeast alcohol dehydrogenase (yADH) at 56 °C. According to differential scanning calorimetry data, 2-HP-β-CD did not affect the stage of the protein molecule unfolding. Dynamic light scattering data indicated changes in the aggregation kinetics of yADH during the nucleation and aggregate growth stages in the presence of the chaperone. The analysis of kinetic curves showed that the order of aggregation with respect to protein (*n*_c_), calculated for the stage of aggregate growth, changed from *n*_c_ = 1 to *n*_c_ = 2 with the addition of 100 mM 2-HP-β-CD. The mechanism of 2-HP-β-CD action on the yADH thermal aggregation leading to a change in its kinetic regime of aggregation is discussed.

## 1. Introduction

**Scheme 1:**
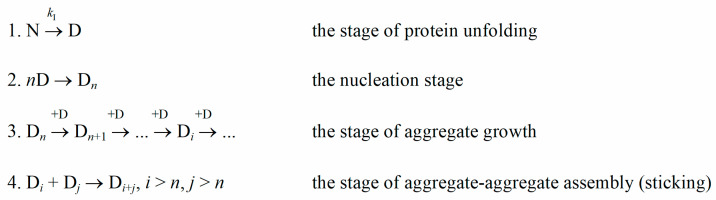
Nucleation-dependent aggregation. N and D are the native and the denatured states of a protein molecule, respectively; *k*_I_ is the rate constant of the monomolecular reaction of a protein molecule unfolding; D*_n_* is a nucleus, consisting of *n* protein molecules.

Protein aggregation is a natural but mostly undesirable process both in vivo and in vitro. Protein aggregates possess a higher light-scattering ability than original protein molecules. Therefore, light-scattering methods are widely used by experimenters to register the kinetics of protein aggregation. Analysis of the kinetic curves of aggregation can provide an understanding of the aggregation stages and changes in their rate under the influence of external factors. When modeling protein aggregation (Scheme 1), the initial stage usually is the stage of the protein molecule unfolding. The presence of a nucleation stage, i.e., the stage of assembling unfolded protein molecules into larger structures (nuclei), is evidenced by the appearance of a concentration-dependent lag phase on the kinetic curves [1,2,3]. The part of the kinetic curve after the lag phase describes the stage of aggregate growth, which is realized by the attachment of unfolded protein molecules to existing nuclei or aggregates. In some cases, there could also be the further stage of aggregate–aggregate sticking [4]. 

Alcohol dehydrogenase I from yeast Saccharomyces cerevisiae (EC 1.1.1.1) is an enzyme catalyzing the reduction of acetaldehyde to ethanol [5]. The molecular mass of yADH is about 150 kDa. It consists of four identical subunits, each containing two zinc ions [5,6]. The catalytic zinc ion in the active site is coordinated by Cys-43, Cys-153, His-66 and water or substrate (supposedly, during the catalytic cycle, the last coordination group changes to the Glu-67 residue) [5]. The conformational zinc ion is coordinated by Cys-97, -100, -103 and -111, it stabilizes the structure of yADH and its active site in particular [6]. Cysteine residue (Cys-277) is also involved in the formation of inter-subunit contacts [5,7]. 

yADH is a very popular model object, especially for enzyme immobilization research, with some of these works directly concerning the problem of protein denaturation during immobilization [8,9,10]. yADH can be used in chemoelectrosensors [11] and biofuel cells [12,13]. Therefore, the studies of the yADH stability and the effects of solution additives can provide useful information for further biotechnological applications. 

It is known [14,15] that the commercial preparations of yADH can contain different amounts of Zn^2+^ per molecule. These preparations also have protein fractions that differ in thermostability while the enzymatic activity of the protein remains sufficiently high for other uses [15]. It was shown that incubation of yADH with dithiothreitol (DTT) reduced thermostability of the protein [6,16] and, in some cases, its activity [7]. To use yADH in experiments involving thermal methods, we should to the extent possible ensure the homogeneity of the target protein in terms of thermostability and propensity to aggregation. It was previously shown that fractions of different stability can be separated by preheating [15]. This pre-heated yADH (yADH_p_) is considered to be the native form with the most intact structure and was used as a model object in the present work. 

Cyclodextrins are cyclic oligosaccharides with a hydrophobic inner cavity. Cyclodextrin molecule consists of several (5–7, usually) glucopyranose subunits linked by α-(1-4) glycoside bonds [17]. 2-hydroxypropyl-β-cyclodextrin (2-HP-β-CD) is a more soluble, substituted form of β-cyclodextrin, consisting of 6 glucopyranose monomers and several 2-hydroxypropil radicals. In this work we used randomly substituted 2-HP-β-CD with the mean substitution degree of 3. Cyclodextrins are used in pharmaceutics and other industrial applications due to relatively low toxicity and the ability to form inclusion complexes with hydrophobic compounds [17,18,19]. This ability includes the interaction with aromatic amino acids in solution [20,21] and in polypeptide chains [22,23], which makes cyclodextrins compounds of interest in protein research. 2-HP-β-CD is widely tested as the agent suppressing protein aggregation. However, the results of such testing often yield controversial results: 2-HP-β-CD can stabilize proteins against various stress conditions (thermal, mechanical stress) and suppress aggregation [24,25,26,27] as well as destabilize proteins and promote aggregation [25,28,29].

In this work, it was shown for the first time that 2-HP-β-CD can change the overall characteristic of the protein aggregation process, i.e., its kinetic regime, and the rate-limiting stage. It added valuable information to our understanding of cyclodextrins as anti-aggregation agents. The determination of the kinetic regime can be a generalized yet precise way to characterize the action of such multifaceted agents as cyclodextrins. 

## 2. Results

### 2.1. Effect of 2-HP-β-CD on the Kinetics of Thermal Aggregation of yADH_p_

**Figure 1:**
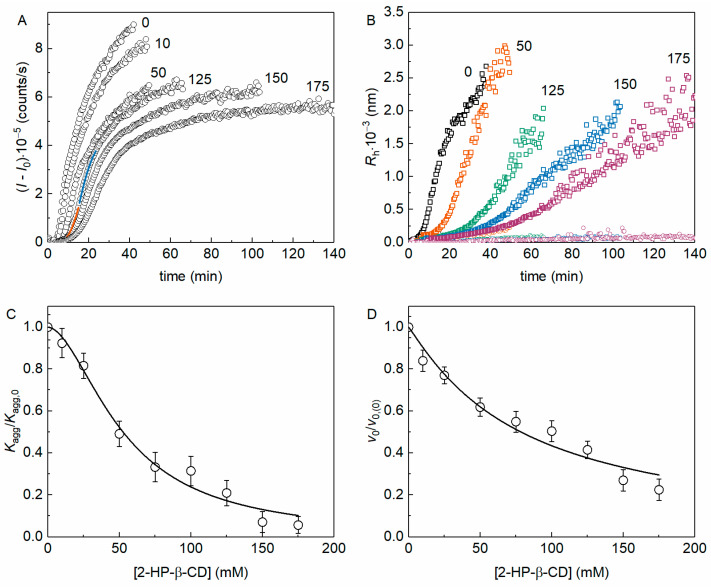
The effect of 2-HP-β-CD on the kinetics of yADH_p_ thermal aggregation at 56 °C (0.4 mg/mL protein, 50 mM Na-phosphate, pH 7.4, 100 mM NaCl). (**A**) Examples of the dependences (*I* − *I*_0_) on time for yADH_p_ in the presence of different 2-HP-β-CD concentrations (indicated in mM on the panel). The points are the experimental data, solid lines are the fittings with Equation (3) (orange curve) and Equation (4) (blue curve). (**B**) Examples of the dependences of the hydrodynamic radius of yADH_p_ aggregates on time in the presence of different 2-HP-β-CD concentrations (indicated in mM on the panel). (**C**,**D**) The dependences of *K*_agg_/*K*_agg,0_ and *v*_0_/*v*_0,(0)_ respectively on 2-HP-β-CD concentration. Points are the experimental data; solid curves were calculated with Equations (9) and (10). The error bars were calculated using three independent measurements.

The kinetics of yADH_p_ thermal aggregation at 56 °C was registered by the increase in the light scattering intensity (*I*) by dynamic light scattering (DLS). Figure 1A shows the kinetic curves of (*I* − *I*_0_), where *I*_0_ is the value of *I* at the initial time, in the presence of different 2-HP-β-CD concentrations (the molar ratio [2-HP-β-CD]/[ADH] varied from 3.8 × 10^3^ to 6.7 × 10^4^). The kinetic curves were analyzed using Equations (3) and (4) as shown in Figure 1A (see Figure 1 legend).

Figure 1B shows the dependences of the hydrodynamic radius of aggregates (*R*_h_) on time (*t*) for different concentrations of 2-HP-β-CD. These *R*_h_(*t*) plots reveal the existence of two populations of particles, as described in our earlier work [15], therefore confirming the previously established mechanism of yADH_p_ aggregation. The populations of aggregates with larger *R*_h_ (*R*_h,1_) are indicated in Figure 1B by squares, whereas the populations with smaller sizes (*R*_h,2_) are indicated by circles of the corresponding color for each concentration of 2-HP-β-CD. The scattering signal obtained by DLS is much more sensitive to bigger particles, so we can suppose that the increase in the *I* value is due to the growth of aggregates with larger *R*_h_. Thus, we can refer to this population as *R*_h_ in the following text.

The initial part of the kinetic curve of aggregation, corresponding to the nucleation stage, can be characterized by the *K*_agg_ parameter (see Equation (3)), which estimates the acceleration of aggregation due to the formation of nuclei from the unfolded protein molecules [30]. It should be noted that for the initial aggregation stage, the relationship between the accumulation of aggregated protein and time squared was also shown by others [31,32]. Figure 1C shows the dependence of the relative acceleration of aggregation *K*_agg_/*K*_agg,0_ on 2-HP-β-CD concentration (*K*_agg,0_ is the value of *K*_agg_ in the absence of the chemical chaperone). This dependence was analyzed with Equation (9). The obtained [L]_0.5_ value can be used as a parameter characterizing the anti-aggregation effectiveness of a chemical chaperone, and for 2-HP-β-CD at this initial stage of yADHp aggregation [L]_0.5_ = 52 ± 3 mM (*h* = 1.8 ± 0.2).

The stage of the yADH_p_ aggregate growth, i.e., the part of the kinetic curve after the inflection point, is characterized by the initial rate of the aggregate growth, *v*_0_. The dependence of the relative rate of aggregate growth in the presence (*v*_0_) and in the absence (*v*_0,(0)_) of the additive on 2-HP-β-CD concentration is shown in Figure 1D. This dependence was analyzed with Equation (10), and parameter [L]_0.5_ for 2-HP-β-CD at the aggregation growth stage was equal to 78 ± 7 mM (*h* = 1.1 ± 0.1).

### 2.2. Effect of HP-β-CD on the Thermostability of yADH_p_

**Figure 2:**
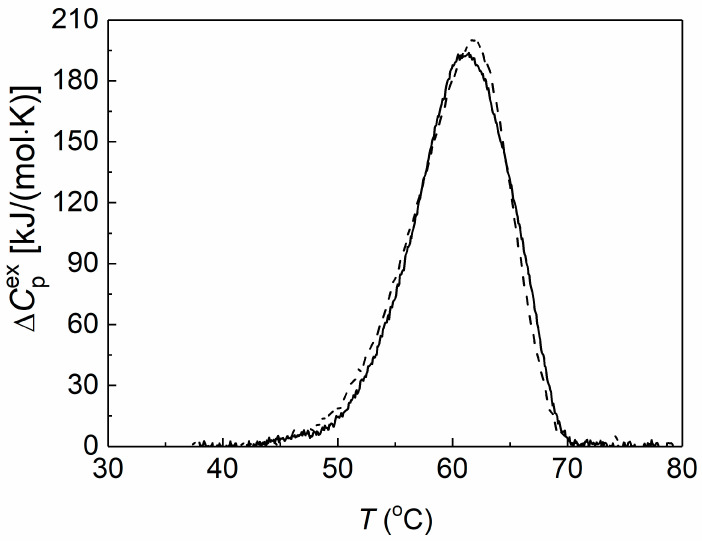
The effect of 2-HP-β-CD on the thermal stability of yADH_p_. The dependences of the excess heat capacity for the thermal denaturation of yADH_p_ (0.73 mg/mL) in the absence (dash line) and in the presence (solid line) of 100 mM 2-HP-β-CD.

Differential scanning calorimetry (DSC) was used to assess the thermal stability of preheated yADH_p_ (0.73 mg/mL) in the absence and in the presence of 2-HP-β-CD. The obtained DSC profiles are shown in Figure 2.

It was shown that the thermal transition maximum (*T*_max_) for yADH_p_ corresponded to the value 61.9 ± 0.1 °C. In the presence of 100 mM 2-HP-β-CD the *T*_max_ position shifted towards the lower temperatures by 0.9 °C to the value 61.0 ± 0.2 °C, which indicates a slight decrease in the yADH_p_ thermal stability. At the same time, the calorimetric enthalpy (Δ*H*_cal_) of the thermal transitions does not change: Δ*H*_cal_ = 2090 ± 104 kJ/mol and 2065 ± 103 kJ/mol in the absence and in the presence of 2-HP-β-CD, respectively. This fact indicates that 2-HP-β-CD does not significantly affect the tertiary structure of the protein molecule.

The denaturation constant, *k*_den_, at 329 K (56 °C) for yADH_p_ in the absence and in the presence of 2-HP-β-CD was determined as described earlier [15]. The *k*_den_ value was 0.060 min^−1^ for yADH_p_ without additive or 0.062 min^−1^ in the presence of 100 mM 2-HP-β-CD.

### 2.3. The Change in the Kinetic Regime of yADH_p_ Aggregation in the Presence of HP-β-CD

**Figure 3:**
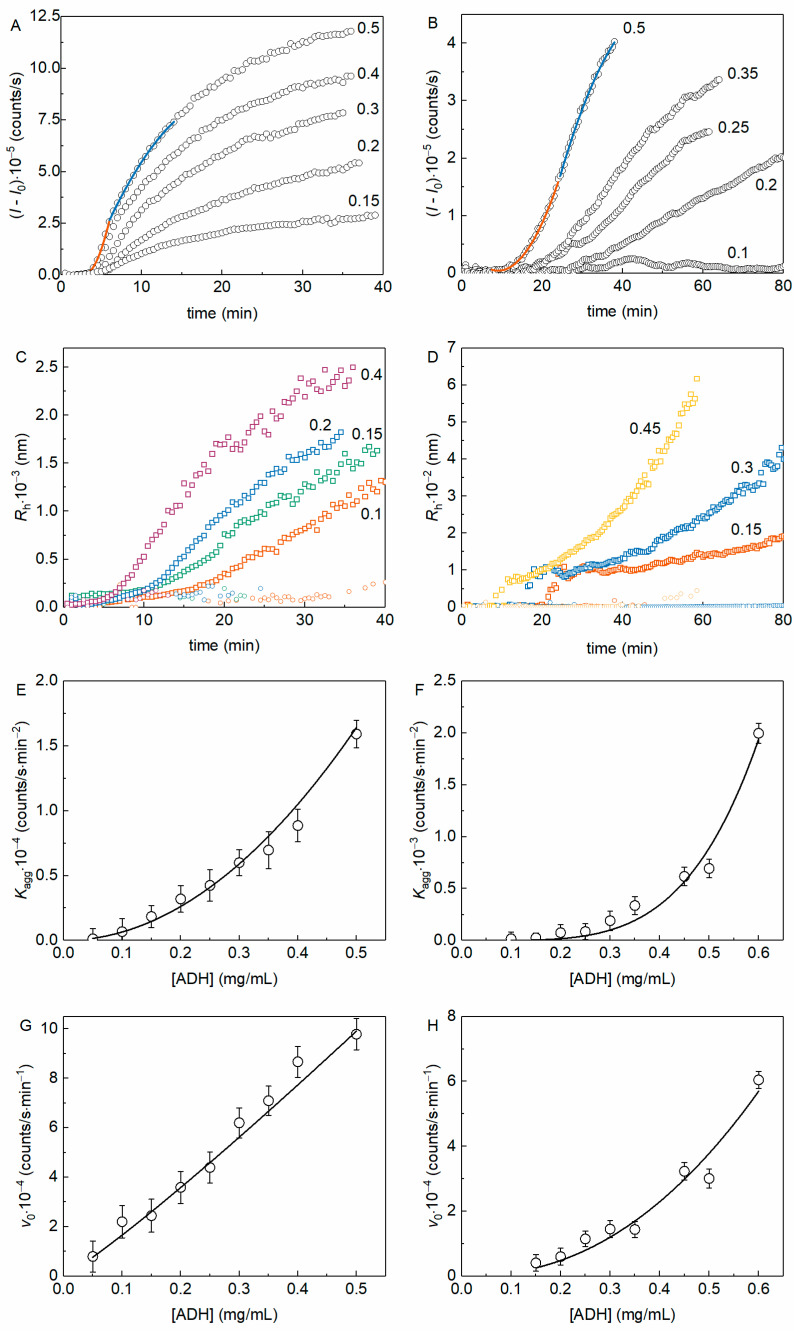
The kinetics of yADH_p_ thermal aggregation at 56 °C in the absence and in the presence of 100 mM 2-HP-β-CD. Examples of the dependences of (*I* − *I*_0_) on *t* in the absence (**A**) and in the presence (**B**) of 100 mM 2-HP-β-CD for different protein concentrations (indicated in mg/mL in each panel). The points are the experimental data, solid lines are the fittings with Equation (3) (orange curve) and Equation (4) (blue curve). Examples of the dependences *R*_h_(*t*) in the absence (**C**) and in the presence (**D**) of 100 mM 2-HP-β-CD for different protein concentrations (indicated in mg/mL in each panel). The dependence of *K*_agg_ on the concentration of yADH_p_ in the absence (**E**) and in the presence (**F**) of 100 mM 2-HP-β-CD. The solid curves were calculated from Equation (5) at the following values of parameters: *const* = 65,659, *b* = 2.1 for the curve on panel 3E and *const* = 17,192, *b* = 4.3 for the curve on panel 3F. The dependence of *v*_0_ on the concentration of yADH_p_ in the absence (**G**) and in the presence (**H**) of 100 mM 2-HP-β-CD. The solid curves were calculated from Equation (6) at the following values of parameters: *const* = 213,890, *b* = 1.1 for the curve on panel 3G and *const* = 179,533, *b* = 2.2 for the curve on panel 3H.

To determine the order of aggregation with respect to the protein (*n*_c_) we analyzed the aggregation kinetic curves obtained at various initial protein concentrations. Figure 3A shows the kinetics of thermal aggregation of yADH_p_ at different protein concentrations (the molar ratio [2-HP-β-CD]/[ADH] varied from 1.5 × 10^3^ to 2.5 × 10^4^), whereas Figure 3B demonstrates equivalent data in the presence of 100 mM 2-HP-β-CD. The examples of fitting of the experimental data by Equations (3) and (4) are also shown in Figure 3A,B (see Figure 3 legend).

Figure 3C,D show the dependences of *R*_h_ on time for yADH_p_ thermal aggregation in the absence and in the presence of 2-HP-β-CD (100 mM). These data also demonstrate the existence of two populations of aggregates in both cases (the population with larger *R*_h_ is designated with squares and the population with smaller *R*_h_ with circles of corresponding color for each protein concentration). It is interesting to note that the relative contribution of the smaller *R*_h_ population to the total light scattering intensity decreases faster in the absence of 2-HP-β-CD, especially at higher protein concentration. The second notable feature of the *R*_h_(*t*) dependences is that the formation of aggregates with bigger *R*_h_ starts with sharp increase after the lag period in the presence of 2-HP-β-CD (see Figure 3D). These two facts should be taken into account for further discussion.

The dependences of the kinetic parameters *K*_agg_ and *v*_0_ on the yADH_p_ concentration are shown in Figure 3E,G (for yADH_p_), and in Figure 3F,H (for yADH_p_ + 100 mM 2-HP-β-CD). The dependences of *K*_agg_ on the protein concentration (Figure 3E,F), characterizing the kinetic regime of aggregation at the nucleation stage, were analyzed using Equation (5). The power coefficient *b* in Equation (5), indicating the order of aggregation, was found to be equal to 2.1 ± 0.1 in the absence and 4.3 ± 0.5 in the presence of 2-HP-β-CD. 

The dependence of *v*_0_ on the yADH_p_ concentration (Figure 3G) in the absence of 2-HP-β-CD is linear. The analysis of the data using Equation (6) gives a value of *b* = 1.11 ± 0.03, indicating that the order of aggregation with respect to the protein, *n*_c_, is equal to 1. In the presence of 2-HP-β-CD (Figure 3H), analysis of the *v*_0_ dependence on protein concentration according to Equation (6) gives *b* = 2.2 ± 0.3, i.e., the order of aggregation becomes equal to 2 (*n*_c_ = 2). The data obtained indicate a change in the kinetic regime of yADH_p_ thermal aggregation in the presence of 2-HP-β-CD.

### 2.4. The Change in the Aggregation Pathway of yADH_p_ in the Presence of 2-HP-β-CD

**Figure 4:**
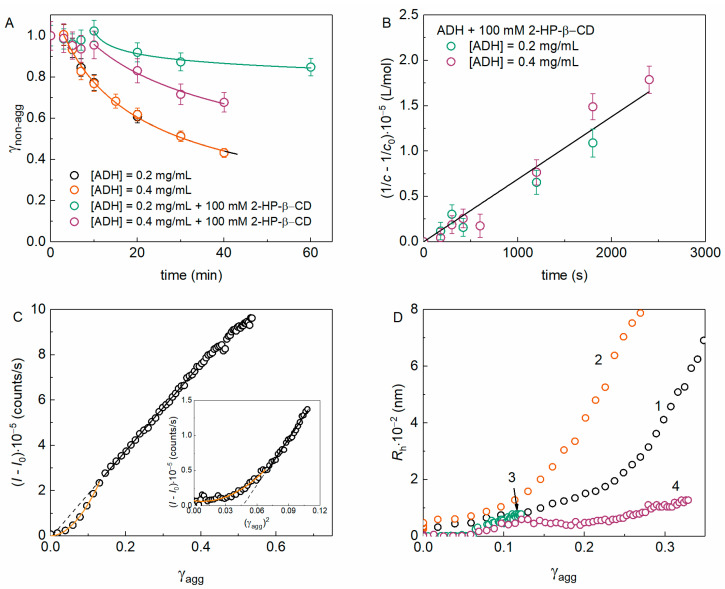
The kinetics of the non-aggregated protein decrease and aggregation pathways for the thermal aggregation of yADH_p_ at 56 °C. (**A**) The dependences γ_non-agg_ (*t*) in the absence and in the presence of 100 mM 2-HP-β-CD. The points are experimental data, solid lines were calculated using Equation (11). The fitting parameters values: *m* = 2.6 ± 0.2, t* = 3.1 ± 0.2 min, *t*_0.5_ = 27.7 ± 0.7 min for yADH_p_ (0.2 or 0.4 mg/mL) in the absence of 2-HP-β-CD; *m* = 20 ± 6, t* = 10.7 ± 0.6 min, *t*_0.5_ = (1.29 ± 0.01)·10^6^ min and *m* = 4 ± 2, t* = 8 ± 2 min, *t*_0.5_ = 88 ± 4 min for 0.2 or 0.4 mg/mL yADH_p_ in the presence of 2-HP-β-CD, respectively. (**B**) The kinetics of yADHp aggregation in the presence of 100 mM 2-HP-β-CD in the coordinates of the second order reaction. (**C**) The relationship between the (*I* − *I*_0_) and the fraction of aggregated protein γ_agg_ (or γ_agg_^2^, inset) for 0.4 mg/mL yADH_p_ in the absence and in the presence of 100 mM 2-HP-β-CD (inset in panel 4C). Orange lines are fittings with quadratic equation, black lines are linear fittings (standard Origin functions were used). (**D**) The relationship between the *R*_h_ of aggregates and γ_agg_ for (1) 0.2 and (2) 0.4 mg/mL yADH_p_ in the absence of 2-HP-β-CD and (3) 0.2 and (4) 0.4 mg/mL yADH_p_ in the presence of 100 mM 2-HP-β-CD.

It is important to supply the light-scattering data with more thorough analysis by the methods giving the direct estimation of the portion of non-aggregated (γ_non-agg_) and aggregated (γ_agg_) protein. The γ_non-agg_ value was measured as described in Section 4.6. Figure 4A shows the dependences of γ_non-agg_ (*t*) for various yADH_p_ concentrations in the absence and in the presence of 100 mM 2-HP-β-CD. It was shown that the decrease in the γ_non-agg_ over time did not depend on the protein concentration (Figure 4A, blue and orange circles), and the kinetic curves for ADH in the absence of 2-HP-β-CD did not differ from each other. This suggests the first order of aggregation with respect to the protein. In the presence of 2-HP-β-CD, the fraction of non-aggregated protein significantly increases, which confirms the suppression of aggregation.

Analysis of the obtained data (Figure 4A) using Equation (11) can provide valuable information about the kinetics of protein aggregation. In addition, this equation can be used for a general description of γ_non-agg_ on time dependences in cases when these data do not obey simpler laws (exponential or linear). As it has been shown in the case of bovine liver glutamate dehydrogenase (GDH) [33], deviations from the first-order kinetics can be a result of the accumulation of non-native protein forms during denaturation and initial stage of aggregation, when aggregates formed from dissociated monomers are indistinguishable in size from the native oligomeric protein. It can be assumed that a similar process also occurs during thermal aggregation of yADH_p_, since it is an oligomeric protein like GDH. 

Since the protein concentration *c* = *c*_0_γ_non-agg_ (where c_0_ is the initial protein concentration) and the order of yADHp aggregation *n*_c_ = 2 in the presence of 2-HP-β-CD (see Section 2.3, Figure 3F,H), then plotting 1/*c* − 1/*c*_0_ = *k*_II_*t* in the coordinates of the second-order reaction (Figure 4B) makes it possible to find the value of the second-order rate constant *k*_II_. For the yADH_p_ in the presence of 2-HP-β-CD *k*_II_ was found equal to 69 ± 3 L∙mol^−1^∙s^−1^.

According to Figure 3C, in the absence of 2-HP-β-CD the dependence of (*I* − *I*_0_) on γ_agg_, which was calculated as γ_agg_ = 1 − γ_non-agg_, is linear at γ_agg_ > 0.15, whereas the initial part at γ_agg_ < 0.15 can be described by a quadratic equation. In the presence of 100 mM 2-HP-β-CD (inset in Figure 4C), the (*I* − *I*_0_) value depends linearly (or squarely at the initial part) on (γ_agg_)^2^, because the aggregation order is two (Figure 4B). The existence of such proportionalities supports the existence of two aggregation stages where the increase in the light scattering intensity is linearly or squarely proportional to the change in the amount of aggregated protein and which can be described by the parameters with quadratic (*K*_agg_, Equation (5)) or linear (*v*_0_, Equation (6)) time dependences.

The dependences *R*_h_(γ_agg_) in the absence and in the presence of 2-HP-β-CD are shown in Figure 4C. These plots indicate that there is a prominent stage of the aggregate–aggregate sticking in the mechanism of the thermal aggregation of yADH_p_. The presence of 100 mM 2-HP-β-CD significantly suppresses this process. The fact that the second, smaller population of aggregates in the presence of 2-HP-β-CD has a more prominent contribution in the total scattering intensity (Figure S1, Supplementary Materials) and at higher concentrations of protein (Figure 1B and Figure 3D) than in the absence of 2-HP-β-CD (Figure 3C) could additionally indicate the suppression of aggregate–aggregate sticking in the presence of the chemical chaperone.

### 2.5. The Zeta Potential of yADH_p_ Aggregates in the Presence and in the Absence of 2-HP-β-CD

To test the ability of 2-HP-β-CD to stabilize protein aggregates against sticking by interfering with electrostatic interactions we measured the zeta-potential of yADH_p_ aggregates formed without chemical chaperone, and in its presence. For the aggregates’ preparation, two protein concentrations were used (0.2 and 0.4 mg/mL). The aggregation times were selected using DLS data to obtain aggregates of similar size (*R*_h_) in the absence and in the presence of 2-HP-β-CD. The zeta-potential values without 2-HP-β-CD were the following: −7.6 ± 0.4 mV for 0.4 mg/mL yADH_p_ and −6.9 ± 0.4 mV for 0.2 mg/mL yADH_p_. In the presence of 100 mM 2-HP-β-CD we observed the decrease in zeta-potential: −1.89 ± 0.05 mV for 0.4 mg/mL yADH_p_ and −1.18 ± 0.01 mV for 0.2 mg/mL yADH_p_. The decrease in the absolute value of zeta potential indicates the decrease in the colloidal stability of the samples and the increase in their propensity to precipitation. Therefore, the electrostatic repulsion cannot explain the suppression of aggregation by 2-HP-β-CD.

### 2.6. The Effect of Solution Viscosity on the Kinetics of yADH_p_ Aggregation

**Figure 5:**
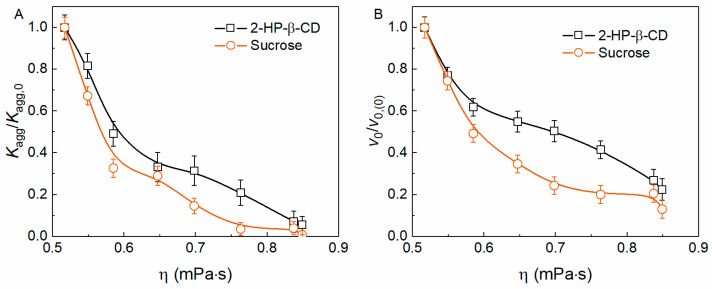
The effect of the solution viscosity on the kinetic parameters of yADH_p_ (0.4 mg/mL) aggregation at 56 °C. The dependences of *K*_agg_/*K*_agg,0_ (**A**) and *v*_0_/*v*_0,(0)_ (**B**) on the solution viscosity at different concentrations of 2-HP-β-CD and sucrose.

One of the most obvious hypotheses about the mechanism of 2-HP-β-CD anti-aggregation activity is the hindering of particle diffusion and interaction by increased solution viscosity. Table S1 (Supplementary Materials) is illustrative of the dynamic viscosity (η) at different concentrations of 2-HP-β-CD. To test the effect of viscosity on yADH_p_ aggregation we compared sucrose’s (Suc) effect on yADH_p_ aggregation kinetics with the effect of 2-HP-β-CD. For this purpose, Suc concentrations with η values equal to those for different concentrations of 2-HP-β-CD (Table S2, Supplementary Materials) were used. The obtained kinetic curves of (*I* − *I*_0_) increase were analyzed as described in Section 2.1 and Section 2.3 using Equations (3) and (4). The dependences of the kinetic parameters *K*_agg_/*K*_agg,0_ and *v*_0_/*v*_0(0)_ on the solution viscosity, η, are given in Figure 5A and Figure 5B, respectively.

It can be seen that 2-HP-β-CD at the same solution viscosity suppresses yADH_p_ aggregation to a lesser extent than Suc, especially at the stage of aggregate growth (Figure 5B). It should also be noted that Suc increases the thermal stability of yADH_p_ compared to 2-HP-β-CD, shifting the position of *T*_max_ by 1.7 °C towards higher temperatures (see Figure S2A, Supplementary Materials). We also have tested the ability of Suc to change the kinetic regime of aggregation at the same viscosity as 100 mM 2-HP-β-CD, which corresponds to 413 mM of Suc (see Table S2, Supplementary Materials). The dependences of *K*_agg_ and *v*_0_ on yADH_p_ concentration were analyzed with Equations (5) and (6) (Figure S2B,C, Supplementary Materials). It was shown that Suc and 2-HP-β-CD have a comparable effect at the same viscosity, and Suc can also change the kinetic regime of aggregation. Therefore, it can be suggested that the solution viscosity plays an important role in the change of the aggregation kinetic regime.

## 3. Discussion

The importance of determining the kinetic regime of aggregation was indicated in our previous works [34,35]. It has been shown that the kinetic regime of aggregation for the same protein can differ depending on conditions [4,36]. In addition, the external influence on the protein structure, in particular, UV irradiation, can also lead to a change in the kinetic regime of its aggregation [35]. In this regard, the question arises: can the presence of chemical chaperone change the kinetic regime in the test system?

Let us assume that protein unfolding is an irreversible monomolecular reaction characterized by a first-order rate constant *k*_I_ (Scheme 1, line 1). The stage of aggregate growth (Scheme 1, line 3) is a bimolecular reaction of addition of denatured monomers D to the formed nuclei D*_n_* and is characterized by a second-order rate constant *k*_II_:

(1) $$D_{n}+D\overset{k_{II}}{\to }D_{n+1}$$

The initial rate of aggregate growth (*v*_0_) expressed as the rate of diminishing concentration of monomers D can be written as follows:

(2) $$v_{0}=-\frac{d\left[D\right]}{dt}=k_{II}\left[D_{n}\right]\left[D\right]$$

The nucleus D*_n_* formed in the nucleation process has a definite number of aggregation points where the attachment of monomer D occurs. If a new aggregation point is produced as the result of the form D attachment, the concentration of nuclei remains constant. In this case, the second-order rate constant *k*_II_ is transformed into the pseudo-first-order rate constant *k*_I_ (*k*_I_ = *k*_II_[D*_n_*]) and order of aggregation with respect to protein (*n*) calculated for the stage of aggregate growth will be equal to unity. Consider the situation when the rate of the attachment of unfolded protein monomers to existing nuclei substantially exceeds the rate of unfolding of the protein molecule. Under these assumptions the rate of aggregate growth is determined by unfolding of the protein molecule, and the pseudo-first order rate constant calculated from the kinetic curve of accumulation of aggregated protein coincides with the first order rate constant *k*_I_ corresponding to the stage of protein unfolding. It is noteworthy that for the kinetic regime of the aggregation process under discussion, the initial rate of aggregation calculated for the stage of aggregate growth is proportional to the initial concentration of the protein. This means that the order of aggregation with respect to protein is equal to unity.

The order of aggregation of yADH_p_ in the absence of additives is determined by varying the protein concentration, *n*_c_ = 1 (Figure 3). This is also indicated by the independence of the γ_non-agg_ kinetic curves from the changes in protein concentration (Figure 4A). These curves deviate from the exponential character of the first order kinetics, apparently due to accumulation of the dissociated denatured protein and small aggregates [33]. The existence of the second, smaller population of aggregates (see Figure 1, Figure 3 and Figure S2) was also confirmed by other works. Particularly, Markossian et al. [15] have shown that a significant portion of yADH remained in soluble forms at 48 °C. According to Fu et al. [37], a fraction of yADH remains in tetrameric soluble form after heating at 50 °C for up to 50 min.

As a rule, interaction with cyclodextrins leads to an increase in the thermal stability of proteins [38,39,40]. However, in some cases, 2-HP-β-CD reduced thermal stability, as was shown for glycogen phosphorylase *b* [40] and glyceraldehyde-3-phosphate dehydrogenase [29]. The present work also showed a slight destabilizing effect of 2-HP-β-CD on yADH_p_ (Figure 2, DSC). Therefore, this chemical chaperone did not prevent the thermal unfolding of the protein molecule (Scheme 1, line 1), and its protective effect was manifested at subsequent stages of the aggregation process. Similar data were obtained for rabbit skeletal muscle creatine kinase [41] and lysozyme [42]: 2-HP-β-CD did not prevent the protein denaturation, but slowed down its aggregation. 

In this work, the following mechanism of yADH_p_ thermal aggregation in the absence and presence of 2-HP-β-CD was proposed (Table 1). In the absence of the additive, the native protein (N) denatures and forms aggregation-prone intermediate (I) [43]. This intermediate forms nuclei/the initial aggregates, I_2_, apparently by dimerization (*n* = 2, see Section 2.3). Strictly speaking, further consequent polymerization (I_2_ + I, or I_2_ + I_2_) can take place, but it could also be considered as a bimolecular reaction. At the following stage of aggregate growth, the addition of newly formed I or I_2_ to the aggregate becomes the main process that occurs quickly. The rate-limiting stage at this point is the monomolecular formation of I (*n*_c_ = 1, Figure 3G, Table 1). The formation of small aggregates (fraction with *R*_h,2_, Figure 3C) can occur, but this fraction is later included in bigger particles (Figure S1, Supplementary Materials). It can be considered as a side reaction of pseudo-first order, therefore not changing the general picture. The aggregate–aggregate sticking also contributes significantly (Figure 4C). 

The presence of 2-HP-β-CD has little effect on the rate of yADH_p_ denaturation, although it slightly destabilizes the structure of the protein molecule, reducing its thermal stability (DSC data). However, 2-HP-β-CD can influence the formation of intermediates (I’, Table 1), probably due to the ability of cyclodextrins to bind to the protein surface [44]. This significantly affects all subsequent stages of the yADH_p_ aggregation process. 

The nucleation stage in the presence of 2-HP-β-CD becomes less favorable and slows down. The aggregation order as determined by the nucleation rate (*K*_agg_) changes from *n* = 2 to *n* = 4 (Figure 3F), which points at the more complex sticking of particles as a limiting process. The similar values of *n* were published earlier for DTT-induced α-lactalbumin aggregation (*n* = 5) [45], where protein denaturation is almost instant [46] and the aggregate-aggregate sticking is, apparently, the main mechanism. In the present work, the sudden appearance of the aggregates with a significant *R*_h_ value after the lag period (Figure 3D), rather than a gradual increase in their size, can also be interpreted as a formation of start aggregates S*_n_* [46] in an “all-or-nothing” manner. It can be assumed that the number of intermediates I’ involved in the formation of the start aggregate is equal to or greater than four (Table 1).

The aggregation of yADH_p_ in the presence of 2-HP-β-CD at the stage of aggregate growth also slows down. The addition of intermediates (I′) to growing aggregates becomes unfavorable, probably due to the presence of protein-bound 2-HP-β-CD, and the aggregates and newly formed nuclei remain closer in size than in the absence of chaperone (Table 1, Agg*_x_* < Agg*_i_*). The size difference of aggregates with *R*_h,1_ and the smaller fraction with *R*_h,2_ also becomes less pronounced (less than two orders of magnitude), so the process of their co-aggregation cannot be considered as the pseudo-first-order kinetics. An increase in the parameter *b* for the dependence *v*_0_([P]) from 1.11 to 2.2 in the presence of 2-HP-β-CD (Figure 3H,G) indicates a change in the order of protein aggregation at the stage of aggregate growth from *n*_c_ = 1 to *n*_c_ = 2. This bimolecular stage of aggregation slows down so much that it becomes the rate-limiting one for the entire aggregation process. It can also be seen (Figure 4C) that presence of 2-HP-β-CD suppresses the aggregate–aggregate sticking. This means that the kinetic regime and the pathway of yADH_p_ thermal aggregation is able to change under the action of 2-HP-β-CD. 

The exact molecular mechanisms by which 2-HP-β-CD and other cyclodextrins suppress protein aggregation were already discussed by many researchers. The proposed mechanisms are the interaction with hydrophobic (especially aromatic) amino acid residues [47,48], the surfactant effects [49,50], hydrogen bonding and water replacement [49]. The increase in the solution viscosity, which is significant at the relatively high concentrations of 2-HP-β-CD [51], could also play a role; however, many studies were performed in the range of 2-HP-β-CD concentrations where the effect of viscosity was not detected [28,47]. 

As mentioned earlier, in some cases cyclodextrins destabilize proteins, supposedly by the same interaction with amino acid residues [52,53,54], and promote protein aggregation [25,55]. It is not uncommon for compounds that are usually regarded as chemical chaperones to have counteracting effects and sometimes promote protein aggregation [4,25,35]. Here, we have shown that 2-HP-β-CD slightly destabilizes the yADH_p_ molecule, as well as reduces the absolute value of the aggregates zeta-potential, therefore lowering the electrostatic repulsion and potentially promoting the sticking and precipitation of aggregates. However, 2-HP-β-CD generally inhibits yADH_p_ aggregation.

The suppression of aggregation in the presence of 2-HP-β-CD may be due to an increase in solution viscosity and its effect on the diffusion and aggregation of particles. Evaluation of the viscosity effect on the aggregation kinetics of yADH_p_ using sucrose showed that most of the determined aggregation kinetic parameters had similar values in the presence of 2-HP-β-CD and sucrose (Figure 5; Figure S2, Supplementary Materials). It can be assumed that the mechanism of action of these agents may be similar. Sucrose can also stabilize proteins and suppress aggregation by preferential exclusion of water [56] and shielding of hydrophobic regions [57], and it should be taken into account when interpreting its effects and comparing them with 2-HP-β-CD.

The more specific mechanism of aggregation suppression in the case of ADH and cyclodextrins was proposed [44] for equine ADH (which is structurally similar enough to yADH [58]) and α-cyclodextrin. It includes the interaction of cyclodextrin with the Phe110 residue in the denatured intermediate and, therefore, decreasing the ability of the latter to form aggregates by consequent polymerization. A similar version was proposed by Starciuc et al. [42], according to which HP-β-CD destabilizes the tertiary structure of lysozyme under heat stress, increasing the flexibility of the protein and causing destabilization of its secondary structure, and inhibition of lysozyme aggregation is associated with the ability of HP-β-CD to form an inclusion complex with the protein and prevent the formation of new strong H-bonds between β-sheet structures. We can suppose that a similar mechanism can be realized in our case for yADH_p_ and 2-HP-β-CD, especially considering the change in the kinetics of the initial aggregation stage from bimolecular to more complex.

Thus, we hypothesize that the protective effect of 2-HP-β-CD on protein stability and aggregation is the sum of different, often counteracting factors. On the one hand, 2-HP-β-CD increases the viscosity of solutions and can prevent yADHp aggregation due to the preferential exclusion of water from the protein surface, like sucrose. On the other hand, direct binding of 2-HP-β-CD to a protein molecule makes aggregate sticking unfavorable. These effects counteract protein destabilization by 2-HP-β-CD and the reduction of electrostatic repulsion, and result in the suppression of aggregation, which is associated with the ability of 2-HP-β-CD to change the kinetic regime of yADHp aggregation. Understanding this, the effects of cyclodextrins on proteins can be interpreted more thoroughly in the future. 

The study of these effects is extremely important for the medical and pharmaceutical applications of cyclodextrins. Cyclodextrins are widely studied as components of drug-delivery systems [59,60], which poses several challenges for researchers. There are various routes of administration of cyclodextrin-based drugs, for example peroral [61], transdermal [62,63], nasal [64], through the eyes [65] and through the lungs [66]. With all these delivery methods, it is necessary to evaluate the possible toxic effects of cyclodextrins [67], including their interaction with cellular components [68] and, in particular, their effects on the proteins’ stability and aggregation in the human body. Another important aspect of research into the effect of cyclodextrins on proteins is the use of cyclodextrins as components of protein and peptide drugs [69,70]. For example, there are a number of works devoted to the effect of cyclodextrins on the aggregation and oligomeric state of insulin [27,71]. It is important to note that protein aggregation studies under a wide range of non-physiological conditions may also be of practical applications. These studies may provide useful information on the aggregation and stability of pharmaceutically relevant proteins during production, storage and transportation. Our work can contribute to methodological approaches and the interpretation of the results of such research.

## 4. Materials and Methods

### 4.1. Materials

yADH (cat. #A3263), monobasic (cat. #S0751) and dibasic (cat. #S9763) sodium phosphate, sodium chloride (cat. #S9888) were purchased from Sigma-Aldrich (St. Louis, MO, USA). 2-HP-β-CD (cat. #CY-2005.1, batch #CYL-2278) was purchased from CycloLab (Budapest, Hungary). Deionized water from Sartorius AriumMini water purification system (Sartorius, Göttingen, Germany) was used for all solutions, buffers and protein preparations.

### 4.2. Purification of the Stable Fraction of yADH

The stable fraction of yADH preparation (yADH_p_) was purified by pre-heating and centrifugation, as described in [15]. The protein concentration was determined spectrophotometrically at 280 nm using Spekol 1300 spectrophotometer (Analytik Jena, Jena, Germany), with the absorbance coefficient *A*^1%^ = 12.6 [59]. The buffer solution used in all experiments was 50 mM Na-phosphate, pH 7.4, 100 mM NaCl.

### 4.3. Differential Scanning Calorimetry (DSC)

Differential scanning calorimetry of yADH_p_ was performed using MicroCal VP-Capillary DSC (Malvern Instruments, Northampton, MA, USA). The heating rate was 1 °C/min. The concentration of yADH_p_ was 0.73 mg/mL. The samples containing 100 mM 2-HP-β-CD were measured against the buffer with the same concentration of 2-HP-β-CD in the control cell. Analysis of DSC data was using MatLab (The MathWorks, Inc., Natick, MA, USA), version R2015a, and Origin (OriginLab Corporation, Northampton, MA, USA), version 8.0 and higher. Correction of calorimetric traces was made by subtracting the instrumental baseline corresponding to the scan with buffer in both cells. The excess heat capacity, Δ*C*_p_^ex^, was calculated per tetramer of yADH with molecular mass of 150 kDa. The temperature at the maximum point of the Δ*C*_p_^ex^ versus temperature profile, *T*_max_, was used to characterize the thermal stability of proteins; calorimetric enthalpy, Δ*H*_cal_, was calculated as the area under the DSC profile.

### 4.4. Dynamic Light Scattering (DLS)

The aggregation kinetics of yADH_p_ at 56 °C was registered using dynamic light scattering measured on a Photocor Complex correlation spectrometer (PhotoCor Instruments, Inc., College Park, MD, USA). The spectrometer was equipped with a He-Ne laser (Model 31-2082, 632.8 nm, 10 mW, Coherent Inc., Santa Clara, CA, USA) and a temperature controller. The scattered light was registered at a 90° angle using a customized algorithm on the base of the Photocor-FC software (v. 7.20.8.128), that allows multiple measurements in a continuous mode. The accumulation time for the autocorrelation function was 30 s. The values of hydrodynamic radius of aggregates (*R*_h_) were calculated using the specialized algorithm based on regularization analysis, which is freely available at https://dls.rogach.org (accessed on 10 September 2020). To determine *R*_h_ values based on the Stokes–Einstein equation, the refractive index (*n*) and dynamic viscosity (η) were measured for each sample (See Section 4.5). 

The initial parts of the kinetic curves, corresponding to the nucleation stage, were analyzed with the empiric equation used earlier in our works [37]:

(3) $$I=I_{0}+K_{agg}{\left(t-t_{0}\right)}^{2},\;\;t>t_{0}$$

where *I* is the light scattering intensity, *I*_0_ is the initial value of *I*, *K*_agg_ is the parameter characterizing the acceleration of aggregation at the nucleation stage, *t*_0_ is the time when the *I* value starts increasing. 

To calculate the initial rate of the aggregation process on the stage of aggregate growth (*v*_0_) and duration of the nucleation stage (t*), the second order polynomial can be used [40]:

(4) $$I=I_{0}+v_{0}\left(t-t\;*\right)-B{\left(t-t\;*\right)}^{2},$$

where t* is a length on the abscissa axis cut off by the theoretical curve calculated with this equation and *B* is a constant.

The dependence of *K*_agg_ on the initial concentration of the protein, [P]_0_, can be characterized by power coefficient *b* [34]:

(5) $$K_{agg}=const{[{P]}_{0}}^{b},$$

Similar dependence of *v*_0_ on [P]_0_ could be described as

(6) $$v_{0}=const{[{P]}_{0}}^{b},$$

This parameter *b* in some cases corresponds to the order of aggregation with respect to the protein, *n*. Therefore, Equation (3) can be used in the following form:

(7) $$K_{agg}=const{[{P]}_{0}}^{n},$$

and Equation (4) in the following form:

(8) $$v_{0}=const{[{P]}_{0}}^{n},$$

for the determination of the kinetic regime and the rate-limiting stage of the protein aggregation by varying the initial protein concentration [P]_0_. 

To describe the dependence of the aggregation rate parameters on the concentration of chemical chaperone, the Hill equation can be used [34]:

(9) $$\frac{K_{agg}}{K_{agg,0}}=\frac{1}{1+{\left(\frac{\left[L\right]}{{\left[L\right]}_{0.5}}\right)}^{h}}\;,$$

or, in the case of the aggregation rate parameter being *v*_0_,

(10) $$\frac{v_{0}}{v_{0,(0)}}=\frac{1}{{1+\left(\frac{\left[L\right]}{{\left[L\right]}_{0.5}}\right)}^{h}}\;,$$

where L is a ligand, *K*_agg,0_ is the value of *K*_agg_ in the absence of ligand, *v*_0,(0)_ is the value of *v*_0_ in the absence of ligand, [L]_0.5_ is the concentration of “semi-saturation”, i.e., the value of [L] at which *K*_agg_/*K*_agg,0_ = 0.5 or *v*_0_/*v*_0_ = 0.5, and *h* is the Hill coefficient. [L]_0.5_ can be considered as a measure of anti-aggregation activity of a chemical chaperone.

### 4.5. Refractometry, Densitometry and Viscosimetry

The refractive index (*n*), the density (ρ) and the dynamic viscosity (η) of the buffer containing different concentrations of 2-HP-β-CD or sucrose were measured using ABBEMAT 500 refractometer (Anton Paar, Graz, Austria), DMA 4500 densitometer (Anton Paar, Graz, Austria) and AMVn microviscometer (Anton Paar, Graz, Austria) with 1.6/1.5 capillary system. The corresponding values of *n*, ρ and η at 56 °C can be found in Supplementary Materials, Table S1.

### 4.6. The Determination of the Portion of Non-Aggregated Protein

The samples containing yADH_p_ and 2-HP-β-CD were heated at 56 °C for different incubation times, then cooled in ice. After that the samples were centrifuged using Eppendorf 5417R centrifuge (Eppendorf, Hamburg, Germany) at 20,000× *g* and 4 °C for 30 min to remove aggregates. The protein concentration was determined as described in Section 4.2. To calculate the portion of the non-aggregated protein (γ_non-agg_), the measured concentrations were related to the initial protein concentration in the non-heated sample: γ_non-agg_ = [yADH_p_]/[yADH_p_]_0_. The dependences of γ_non-agg_ were analyzed using the following equation [33]:

(11) γnon−agg=11+2m−1−1t−t*t0.51m−1 , (t>t*)

where t* is the duration of lag period, *t*_0.5_ is the time when γ_non-agg_ = 0.5, *m* is the parameter which describes the shape of the kinetic curve. 

### 4.7. Zeta Potential Measurements

Zeta potential of yADH_p_ aggregates was measured using the Photocor Compact-Z instrument (Photocor Instruments, Inc., College Park, MD, USA). Laser with a wave-length of 654 nm was used as a light source. The measurements were conducted at electrical field voltage 5 V/cm and 23 °C in cylindrical glass vials with Au electrodes. The distance between electrodes was 0.4 cm. The scattered light was collected at a 20° angle. The accumulation time for the autocorrelation function was 30–60 s. All measurements were repeated ten times for each sample.

### 4.8. Data Analysis

All data were analyzed using Origin software (OriginLab Corporation, Northampton, MA, USA) of version 8.0 and higher. The coefficient of determination *R*^2^ characterized the fittings of the experimental kinetic data with the equations used.

## 5. Conclusions

When studying chemical chaperones, the complexity of their action often comes to the fore, including both physicochemical effects and interaction with the target protein. Determining the kinetic regime of aggregation of a model protein can serve as a descriptive tool to characterize the overall effect of the chaperone on it. This work demonstrates for the first time the possibility of changing the kinetic regime of protein aggregation in the presence of a chemical chaperone. It was established that in the presence of 2-HP-β-CD, the limiting stage of thermal aggregation of ADH at 56 °C becomes the stage of growth of aggregates instead of the stage of denaturation of the protein molecule and the order of protein aggregation changes from *n*_c_ = 1 to *n*_c_ = 2. The protective effect of 2-HP- β-CD on ADH aggregation is due to both an increase in solution viscosity and the direct interaction of cyclodextrin with a protein molecule, which together makes the adhesion of unfolded protein molecules and aggregates unfavorable. The data obtained and the approaches used in the work can be useful to other researchers working on the problems of protein aggregation and its prevention.

## Figures and Tables

**Table 1:** The proposed mechanism of yADH_p_ aggregation in the absence and in the presence of 2-HP-β-CD.

Stage of Aggregation	yADH_p_	yADH_p_ + HPCD
1. Denaturation	Formation of intermediates, N→I ***rate-limiting stage***	Formation of intermediates, N→I′ *slight effect of HPCD on yADH_p_ thermal stability*
2. Nucleation	Formation of nuclei by dimerization of intermediates, I + I→I_2_ *fast stage*	Formation of start aggregates, *n*I′→S*_n_*, *n* ≥ 4 *unfavorable process, slow stage*
3. Aggregate growth	Addition of *I* or *I*_2_ to the nuclei, (I + I_2_) + I_2_→Agg*_i_* *fast stage, n_c_ =* 1	Slow addition of intermediates to start aggregates, I′ + S*_n_* →Agg*_x_*, *x* < *i* ***rate-limiting stage**, n_c_ =* 2
4. Sticking	Aggregates sticking, Agg*_i_* + Agg*_j_*→Agg*_i_*_+*j*_	No sticking

N is the native protein, I and I′ are the denatured intermediates of yADH_p_, S*_n_* is the start aggregate, Agg is the aggregate consisting of different numbers of protein molecules (*x,i,j*).

## Data Availability

Data are contained within the article and Supplementary Materials.

## Supplementary Materials

The following supporting information can be downloaded at: https://www.mdpi.com/article/10.3390/ijms242216140/s1.
