# Supplementary material for: Change in the Kinetic Regime of Aggregation of Yeast Alcohol Dehydrogenase in the Presence of 2-Hydroxypropyl-β-cyclodextrin
Source: Int J Mol Sci. 2023 Nov 9;24(22):16140. doi: 10.3390/ijms242216140 (PMC10671268; doi:10.3390/ijms242216140)
Supplement: Supplementary file 1 [file ijms-24-16140-s001.zip › ijms-2680894-supplementary.pdf]

International Journal of
Molecular Sciences

MDPI

Supplementary Materials

Change in the Kinetic Regime of Aggregation of Yeast Alcohol Dehydrogenase in the Presence of 2-Hydroxypropyl-β-cyclodextrin

Vera A. Borzova *, Andrey M. Chernikov, Valeriya V. Mikhaylova and Boris I. Kurganov †

Bach Institute of Biochemistry, Federal Research Centre "Fundamentals of Biotechnology" of the Russian Academy of Sciences, Leninsky pr. 33, 119071 Moscow, Russia; chernikov.andrei.m@gmail.com (A.M.C.); mikhaylova.inbi@inbox.ru (V.V.M.); boris@kurganov.com (B.I.K.)

* Correspondence: vera.a.borzova@gmail.com

† Deceased.

1. The kinetics of different populations of yADH$_P$ aggregates in the absence and in the presence of 2-HP-β-CD

The analysis of aggregation kinetic curves obtained by DLS allowed us to track the formation and disappearance of different populations of protein aggregates. Peak area in Figure S1 corresponds to the contribution of different aggregation species to the total light scattering intensity (calculated from the Intensity distribution).

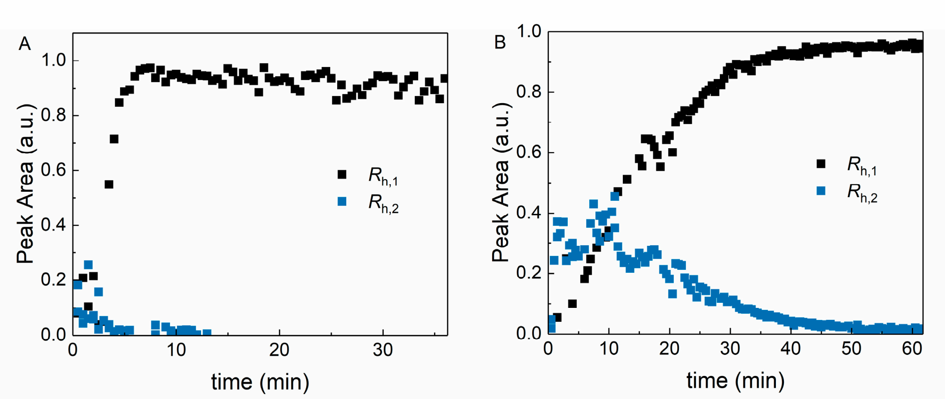

Figure S1. The behavior of two main populations of yADHp aggregates (50 mM Na-phosphate buffer, pH 7.4, 100 mM NaCl, 56 °C, [yADHp] = 0.4 mg/mL) in the absence (A) and in the presence (B) of 100 mM 2-HP-β-CD.

2. Determination of refractive index, density and dynamic viscosity of 2-HP-β-CD solutions

Table S1. The values of refractive index (*n*), density (ϱ) and dynamic viscosity (η) of 2-HP-β-CD in 50 mM Na-phosphate buffer (pH 7.4, 100 mM NaCl) at 56 °C.

[2-HP-β-CD] (mM)	*n*	ϱ (g/cm^3)	η (mPa·s)
0	1.32996 ± 0.00002	0.9948 ± 0.0005	0.517 ± 0.001
25	1.33324 ± 0.00002	1.0021 ± 0.0005	0.549 ± 0.001
50	1.33652 ± 0.00002	1.0092 ± 0.0005	0.585 ± 0.001
75	1.34110 ± 0.00002	1.0191 ± 0.0005	0.647 ± 0.003
100	1.34422 ± 0.00002	1.0268 ± 0.0005	0.698 ± 0.001
125	1.34796 ± 0.00002	1.0348 ± 0.0005	0.763 ± 0.003
150	1.35145 ± 0.00002	1.0426 ± 0.0005	0.837 ± 0.004
175	1.35230 ± 0.00002	1.0443 ± 0.0005	0.8493 ± 0.0001

3. The effect of solution viscosity on thermal aggregation of yADH$_P$

To estimate the role of the solution viscosity in the suppression of yADH$_P$ thermal aggregation, we measured the aggregation kinetics of yADH$_P$ in the solutions of sucrose. Its concentrations were chosen to correspond to the viscosity of 2-HP-β-CD solutions from Table S1.

Table S2. The concentrations of 2-HP-β-CD and sucrose with corresponding values of viscosity (η) in 50 mM Na-phosphate buffer (pH 7.4, 100 mM NaCl) at 56 °C.

η (mPa*s)	[2-HP-β-CD] (mM)	[Sucrose] (mM)
0.517 ± 0.001	0	0
0.549 ± 0.001	25	81
0.585 ± 0.001	50	183
0.647 ± 0.003	75	320
0.698 ± 0.001	100	413
0.763 ± 0.003	125	517
0.837 ± 0.004	150	620
0.8493 ± 0.0001	175	637

To compare the effects of sucrose on the thermostability and aggregation kinetic regime of yADHp with those of 100 mM 2-HP-β-CD, the concentration of sucrose 413 mM was chosen (its viscosity corresponds to that of 100 mM 2-HP-β-CD).

The effect of sucrose on the thermal stability of yADHp studied by DSC in the same conditions as in the case of 2-HP-β-CD ([yADH$_P$] = 0.73 mg/mL, 50 mM Na-phosphate buffer, pH 7.4, 100 mM NaCl, 56 °C, scanning rate 1 °C/min) is shown in Figure S2A.

The aggregation kinetics of yADH$_P$ in the presence of sucrose was measured by DLS in the same conditions as in the case of 2-HP-β-CD ([yADH$_P$] = 0.4 mg/mL, 50 mM Na-phosphate buffer, pH 7.4, 100 mM NaCl, 56 °C). The kinetic curves of light scattering intensity were analyzed with Eq. (3) and (4). The obtained values of the aggregation process parameters are presented in Figure S2.

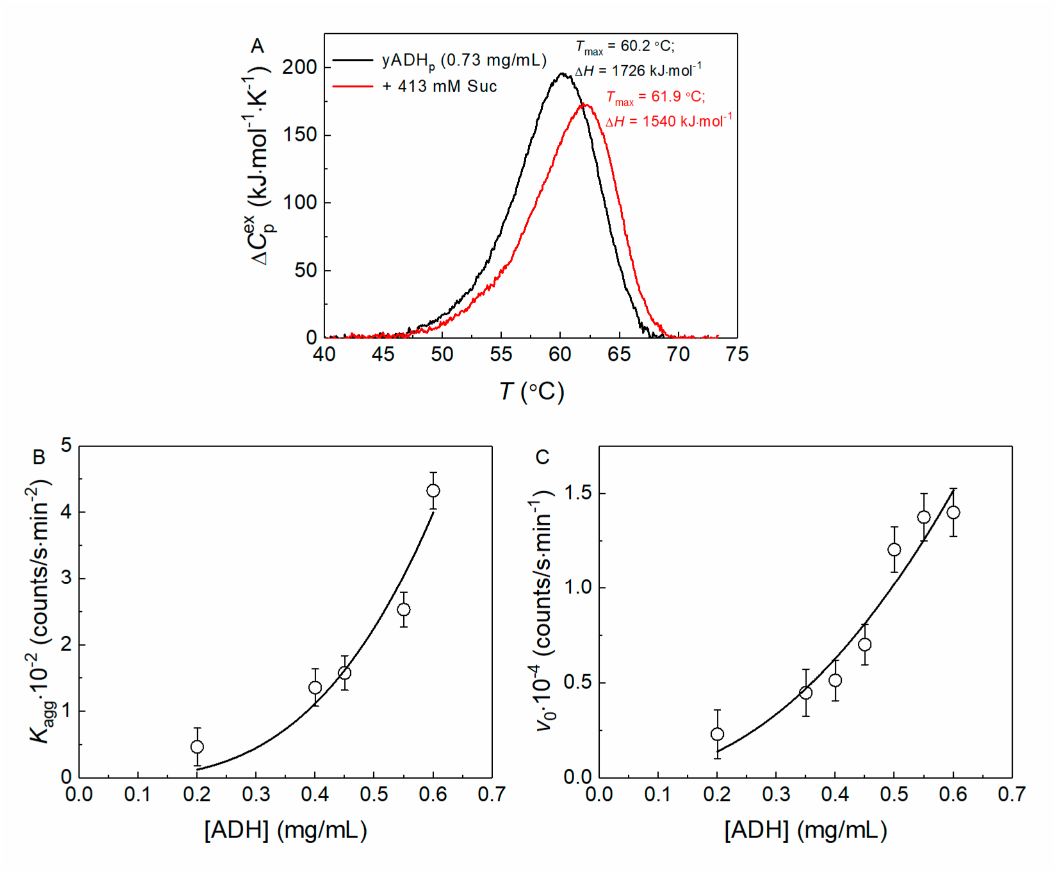

Figure S2. The effect of Suc on the thermostability and aggregation kinetics of yADH$_P$. (A) The dependences of the excess heat capacity for the thermal denaturation of yADH$_P$ (0.73 mg/mL) in the absence and in the presence of 413 mM Suc. (B) The dependence of K_{agg} on the concentration of yADH$_P$ in the presence of 413 mM Suc. (C) The dependence of v_0 on the concentration of yADH$_P$ in the presence of 413 mM Suc.

The dependences of K_{agg} and v_0 on protein concentration were analyzed with Eq. (5) and (6), respectively. The following values of parameter b were obtained: b = 3.1 ± 0.7 for the nucleation stage (Figure S2B) and b = 2.2 ± 0.4 for the aggregates growth stage (Figure S2C).
